# Characterization of lncRNA-Associated ceRNA Network to Reveal Potential Prognostic Biomarkers in Lung Adenocarcinoma

**DOI:** 10.3389/fbioe.2020.00266

**Published:** 2020-04-17

**Authors:** Yang Wang, Ruyi He, Lixin Ma

**Affiliations:** State Key Laboratory of Biocatalysis and Enzyme Engineering, Hubei Key Laboratory of Industrial Biotechnology, Hubei University, Wuhan, China

**Keywords:** lung adenocarcinoma, signature, ceRNA, lncRNA, prognosis

## Abstract

Lung adenocarcinoma (LUAD) is one of the most fatal malignant tumors harmful to human health. The complexity and behavior characteristics of long-non-coding RNA (lncRNA)-associated competing endogenous RNA (ceRNA) network in LUAD patients are still unclear. The purpose of this study was to elucidate the regulatory networks of dysregulated RNAs, view, and identify potential prognosis signatures involved in LUAD. The expression profiles of mRNAs, lncRNAs, and miRNAs were obtained from the TCGA database. In total, 2078 DEmRNAs, 257 DElncRNAs, and 101 DEmiRNAs were sorted out. A PPI network including 45 DEmRNAs was constructed. Ten hub genes in the PPI network associated with cell cycle-related pathways were identified and they played key roles in regulating cell proliferation. A total of three DEmiRNAs, seven DElncRNAs, and six DEmRNAs were enrolled in the ceRNA network. Except for certain genes without any published study reports, all the genes in the ceRNA network played an essential role in controlling tumor cell proliferation and were associated with prognosis in LUAD. Finally, based on step regression and Cox regression survival analysis, we identified four candidate biomarkers, including miR490, miR1293, LINC01740, and IGF2BP1, and established a risk model based on the four genes. Our study provided a global view and systematic dissection of the lncRNA-associated ceRNA network, and the identified four genes might be novel important prognostic factors involved in LUAD pathogenesis.

## Introduction

Despite medical advances, lung cancer remains the leading cause of cancer deaths. Lung cancer is usually recognized late in its natural history and have a poor prognosis, with an overall 5-year survival rate of 10–15% (Cagle et al., 2013). The recognition of histologic subtypes of non-small cell lung carcinoma (NSCLC), namely, adenocarcinoma, squamous cell carcinoma, and large cell lung carcinoma as the most frequent subtypes, has become important as a determinant of therapy in this disease (Kerr et al., 2014). In addition, in recent years, the identification of molecular abnormalities in a large proportion of patients with lung cancer has allowed the emergence of personalized targeted therapies and has opened new horizons and created new expectations for these patients (Ezeife and Leighl, 2018). The use of predictive biomarkers to identify tumors that could respond to targeted therapies has meant a change in the paradigm of lung cancer diagnosis (Majeed and Amir, 2018).

Currently, the rapid advancement of high-throughput technologies offers great opportunities for biomarker identification (Yu et al., 2018). Non-coding RNAs as biomarker and therapeutic targets play a significant role in human disease (Zhou et al., 2018b, c). Among which, long-non-coding RNA (lncRNAs) are a class of RNA molecules with more than 200 nucleotides in length and have no evident open reading frames (Fatica and Bozzoni, 2014). These long molecules are dysregulated among cancers (Yan et al., 2015) and play key roles in gene regulation and carcinogenesis, including proliferation, survival, migration, and genomic stability (Gutschner et al., 2013; Castro-Oropeza et al., 2018). It is believed that the clinical value of lncRNA is not confined to candidate biomarkers for diagnostic and prognostic purposes (Shi et al., 2018).

In Salmena et al. (2011) put forward a competing endogenous RNA (ceRNA) hypothesis. Subsequently, several studies also mentioned that there is an interplay between lncRNAs and miRNAs during the tumorigenic process, among which lncRNAs serve as molecular sponges for miRNAs (Liz and Esteller, 2016). For example, KCNQ1OT1 promotes cell proliferation and autophagy and inhibits cell apoptosis via regulating miR204-5p/ATG3 axis, providing a promising target for NSCLC therapy (Kang et al., 2019). Guo et al. reported that LINC00173 up-regulated Etk through functioning as a ceRNA by “sponging” miRNA-218 and led to the up-regulation of GSKIP and NDRG1 in small cell lung cancer (Zeng et al., 2019). LncRNA AGAP2-AS1 up-regulates ANXA11 expression by sponging miR16-5p and promotes proliferation and metastasis in hepatocellular carcinoma (Liu et al., 2019). Thus, the discovery of lncRNA–miRNA–mRNA networks may lead to a more comprehensive understanding of the etiology and metastasis mechanism of cancer. However, the complexity and behavior of lncRNA-associated ceRNA network remain poorly characterized in lung adenocarcinoma (LUAD).

In this study, by comprehensively integrating gene and miRNA expression data of LUAD, the LUAD-related lncRNA–miRNA–mRNA competitive network was established. We analyzed and predicted the functions of ceRNA and PPI networks and established a Cox regression model to predict the overall survival of patients with lung cancer. Finally, four predictive genes were identified, including LINC01740, mir1293, mir490, and IGF2BP1, which could contribute to LUAD. This study will contribute to understanding the molecular mechanism and provide new therapeutic targets for LUAD.

## Materials and Methods

### Data Preparation and Differentially Expressed Gene Analysis

All primitive data of LUAD from The Cancer Genome Atlas (TCGA) database^1^ were download through GDC Data Transfer Tool, including RNA-seq and miRNA-seq of Transcriptome profiling and Clinical data. EdgeR package (3.3.3 version) (Robinson et al., 2010) in R software was used to analyze and identify differentially expressed RNAs (DERNAs, including DEmRNAs and DElncRNAs) and differentially expressed microRNA (DEmiRNAs) with the thresholds of | log2FoldChange| > 2.0 and FDR (adjusted *p* value) < 0.01. Then, biomart in R package was used to annotate DEmRNAs and DElncRNAs. The heatmap and volcano plot were constructed by the ggplot2 package in R software (Zhou et al., 2017).

### Functional Enrichment Analysis

clusterProfiler (Yu et al., 2012) package in R was used to make the Kyoto Encyclopedia of Genes and Genomes (KEGG) pathway and Gene Ontology (GO) enrichment analysis, including biological process (BP), the cellular component (CC), and molecular function (MF). Pathview (Luo and Brouwer, 2013) and enrichplot packages (Ito and Murphy, 2013) were used to visualize the enrichment results. A significance level of adjusted *p* < 0.05 was set as the cutoff criteria.

### Protein–Protein Interaction Analysis

The DEmRNAs were enrolled in a protein–protein interaction (PPI) network through the STRING (version 11.0) database^2^ with a confidence score >0.9. Furthermore, genes with degree ≥25 were selected as hub genes, and we focused the interaction types among proteins only on physical interaction and co-expression (Sun et al., 2019). Subsequently, GO and KEGG analyses of the PPI network modules were carried out using clusterProfiler package in R.

### Construction of the ceRNA Network

According to the hypothesis of ceRNA, a lncRNA–miRNA–mRNA network was constructed (Zhou et al., 2018a). Relevant miRNA-target data were obtained from the miRTarBase, and the support types of targeting were only focused on experiments, including luciferase reporter assay, Western blot, Northern blot, or qRT-PCR. Only the miRNA targets that were differentially expressed between tumor and normal tissue were considered for the next analysis step. Furthermore, the candidate DElnRNA–DEmiRNA interactions were selected based on miRcode database and the following model:

$$Y_{\text{target}}=$$

(1) $$\beta_{0}+\beta_{1}*m\,i\,R\,N\,A+\beta_{2}*l\,n\,c\,R\,N\,A+\beta_{3}*m\,i\,R\,N\,A*l\,n\,c\,R\,N\,A+\varepsilon$$

where*miRNA*, *lncRNA*, and *Y*_target_ are the gene expression of miRNA, lncRNA, and miRNA targets, respectively. *β*_1_and *β*_2_ represent the effect of miRNA and lncRNA, respectively, on target by themselves alone (main effects), while *β*_3_ represents the effect of miRNA–lncRNA interaction. If a lncRNA and miRNA interaction has effects on target expression outcomes, we expect *β*_3_to be non-zero.Here, all the miRNAs andlncRNAs and miRNA targets should be differentially expressed between tumor and normal tissue.

### Biomarkers Screening and Validation

The status and survival time of LUAD patients were extracted from the TCGA clinical dataset. Subsequently, the DEmRNAs, DElncRNAs, and DEmiRNAs identified in ceRNAs were selected for screening biomarkers. We used univariate Cox regression to screen prognostic factors (*p* < 0.05), and those prognostic factors whose expression levels were significantly relevant to patients’ overall survival (*p* < 0.05) were selected as primitive biomarkers (Zhou et al., 2018b; Bao et al., 2019).

### Cox Risk Regression Establishment and Validation

The lncRNAs, mRNAs, and miRNAs raw data were transformed and normalized in a log2[cpm(*x*) + 1] manner. Univariate cox regression was used to select prognosis-associated genes (*p* < 0.05) (Zhou et al., 2018a). Subsequently, we performed Cox regression analysis combined with stepwise regression to establish a Cox risk model (Zhou et al., 2018a). Finally, a validation set and Kaplan–Meier survival curves along with a logrank *p* test were applied to validate its accuracy (Zhou et al., 2017; Sun et al., 2019).

## Results

### Identification of Differentially Expressed Genes

RNA expression profiles and corresponding clinical data of 533 cohort LUAD patients and 59 normal controls were downloaded from the TCGA database. Meanwhile, miRNA-seq data corresponding to 561 patients’ clinical information, including 515 cohort LUAD patients and 46 normal controls, were obtained from TCGA. In total, 60,483 transcripts and 1046 miRNAs were obtained. With the cutoff criteria unified, CPM(gene) > 1, rowSum(CPM) ≥ 2, 32,495 transcripts and 613 miRNAs were selected for the differentially expressed analysis. After filtering, 5624 DERNAs and 673 DEmiRNAs were identified with the thresholds of | log2FoldChange| > 2.0 and FDR (adjusted *p* value) < 0.01.

**FIGURE 1:**
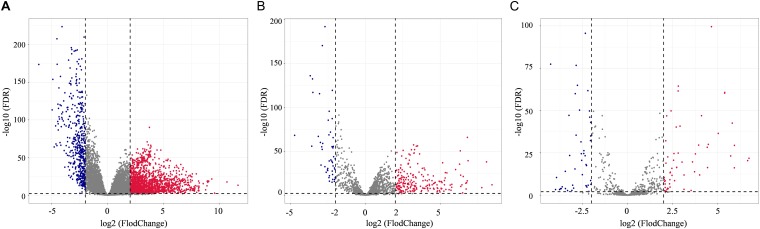
Distribution of differentially expressed genes in lung adenocarcinoma (LUAD) (| log2FoldChange| > 2.0 and adjusted *p* value < 0.01) between 533 tumor tissues and 59 normal tissues. The volcano plots described 2378 DEmRNAs **(A)**, 357 DElncRNAs **(B)**, and 101 DEmiRNAs **(C)**. Red stands for up-regulations, blue stands for down-regulations, and gray stands for normal expression in volcanoes. Each point represents a gene.

In total, 2078 DEmRNAs (1612 up-regulated and 466 down-regulated, Figure 1A), 257 DElncRNAs (209 up-regulated and 48 down-regulated, Figure 1B), and 101 DEmiRNAs (56 up-regulated and 45 down-regulated, Figure 1C) were sorted out.

### Functional Analysis of DERNAs

**FIGURE 2:**
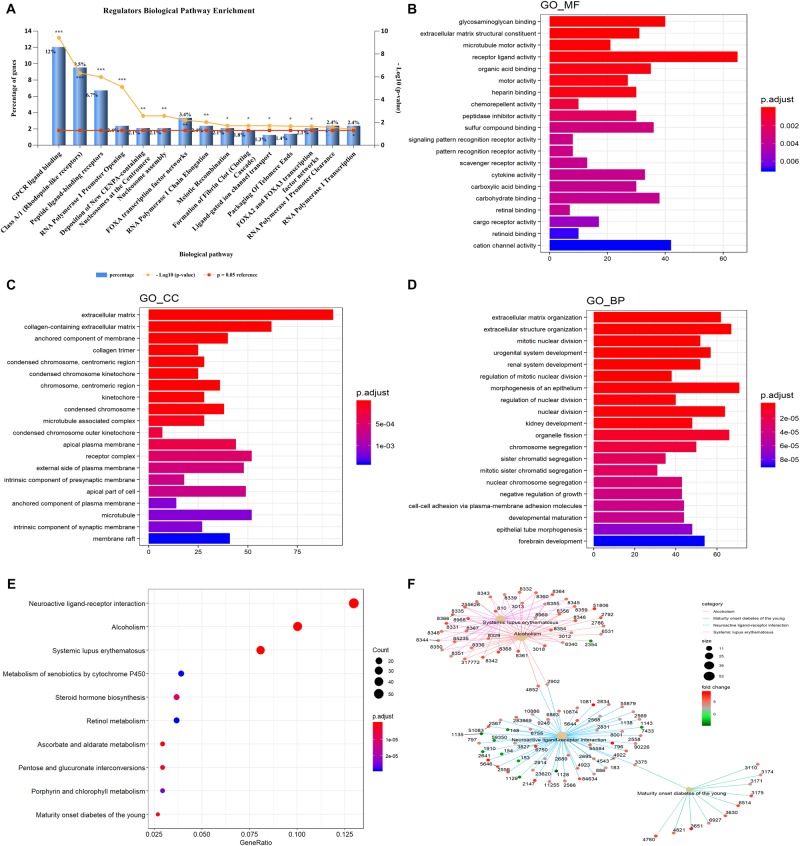
GO and KEGG pathway enrichment analysis of DERNAs. **(A)** The statistical results of genes enriched in biological pathways. The *y* axis on the left represents the percentage of genes in each biological pathway, the *y* axis on the right is –log10(*p* value) of each enrichment pathway, and the *x* axis represents the pathways categories. GO analysis contains the molecular function **(B)**, cellular component **(C)**, biological process **(D)**, the *y* axis represents the number of target genes, and the *x* axis represents the GO categories. **(E)** The most important KEGG pathways in DERNAs. The *y* axis represents the pathways, and the *x* axis represents enriched gene numbers. The circle size represents the counts of genes in each pathway and the color means adjusted *p* value. **(F)** The netplot of KEGG pathways means enrichment of genes in different pathways. The number adjacent to nodes stands for gene ID. The color bar represents the fold change of genes in different pathways. *p < 0.05, **p < 0.005, and ***p < 0.0005.

Gene ontology and KEGG enrichment analyses were used to explore the potential function of DERNAs. Ten biological pathways were highly enriched within cutoff *p* value < 0.05. Among them, 12% DERNAs were enriched in GPCR ligand binding process, and 9.5% DERNAs were enriched in Class A/1 (Rhodopsin-like receptors) pathway, and 6.7% DERNAs were enriched in peptide ligand binding receptor pathways (Figure 2A). Detailed information of these enriched pathways and associated genes is summarized in Table 1. The GO functional enrichment analysis results of DERNAs including MF, CC, and BP were described in Figures 2B–D. The results show that the genes mainly focused on receptor ligand activity function, extracellular matrix, and morphogenesis of an epithelium process.

**TABLE 1:** Enriched biological pathways and associated genes.

**Biological pathway**	**No. of genes in DERNAs**	**No. of genes in background dataset**	**Percentage of genes**	**Fold enrichment**	***p* value**	***p*-adjusted (Bonferroni)**
GPCR ligand binding	86	336	12.0	2.2	5.05E-14	8.42E-11
Class A/1 (rhodopsin-like receptors)	68	274	9.5	2.1	1.26E-10	2.1E-07
Peptide ligand-binding receptors	48	167	6.7	2.5	4.13E-10	6.89E-07
RNA polymerase I promoter opening	17	31	2.3	4.8	4.09E-09	6.83E-06
Deposition of new CENPA-containing nucleosomes at the centromere	15	35	2.1	3.7	2.11E-06	0.003
Nucleosome assembly	15	35	2.1	3.7	2.11E-06	0.003
FOXA transcription factor networks	24	81	3.3	2.6	5.93E-06	0.009
RNA polymerase I chain elongation	17	48	2.3	3.1	1.00E-05	0.016
Meiotic recombination	15	42	2.1	3.1	2.99E-05	0.049
Formation of fibrin clot (clotting cascade)	13	33	1.8	3.4	3.05E-05	0.051
Ligand-gated ion channel transport	9	17	1.2	4.6	3.11E-05	0.051
Packaging of telomere ends	10	21	1.4	4.1	3.65E-05	0.060
FOXA2 and FOXA3 transcription factor networks	15	43	2.1	3.0	4.12E-05	0.068
RNA polymerase I promoter clearance	17	54	2.3	2.7	5.79E-05	0.096
RNA polymerase I transcription	17	56	2.3	2.6	9.67E-05	0.161

Furthermore, KEGG pathway enrichment analysis results demonstrated that the most significantly enriched pathways were neuroactive ligand–receptor interaction, alcoholism, and systemic lupus erythematosus pathways (Figure 2E). The pathway–pathway interaction network (PPIN) was constructed based on the DERNAs enriched in same pathway (Figure 2F). Four pathways were identified in the PPIN, including alcoholism, maturity onset diabetes of the young, neuroactive ligand–receptor interaction, and systemic lupus erythematosus pathway. We noticed that, all the DERNAs enriched in systemic lupus erythematosus and alcoholism pathways were up-regulated, except for gene 2354, whose gene symbol is “FOSB.” Gene annotation of FOSB shows that it was a proto-oncogene, and it has been implicated as regulators of cell proliferation, differentiation, and transformation. Similarly, all the genes enriched in maturity onset diabetes of young pathway were all up-regulated in LUAD. Furthermore, the results showed that gene 4852 and 2092 were both enriched in alcoholism pathway and neuroactive ligand–receptor interaction pathway, and played vital roles in connecting the two pathways.

### PPI Network Analysis

**FIGURE 3:**
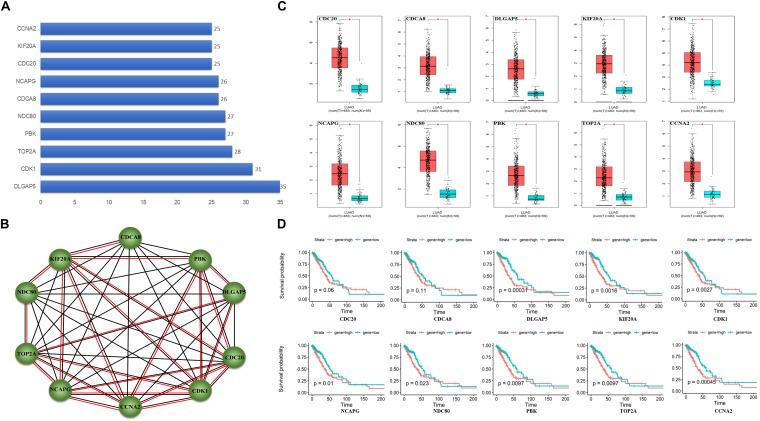
Protein–protein interaction (PPI) network analysis. **(A)** Ten hub genes in PPI based on the DEmRNAs with a combined score of >0.9 and degree ≥25. **(B)** Ten hub genes interaction network. Circles indicate the genes in the PPI network, and the connection indicates the potential interaction between different mRNAs. The red line means physical interaction, and the black line means co-expression with each other. **(C)** Gene expression of 10 hub genes between LUAD tumor and normal tissues. **(D)** Overall survival curves of the 10 hub genes in LUAD. *p < 0.05.

A total of 55 proteins and 453 edges, including 45 DEmRNAs, were selected in the PPI network. A total of 10 hub genes, including CDK1, TOP2A, PBK, CDCA8, CDC20, KIF20A, DLGAP5, NDC80, NCAPG, and CCNA2, were selected from the PPI network with degree ≥25 and combined score >0.9 (Figure 3A). Furthermore, the association among these interacted proteins should be physical interaction or co-expressed with each other (Figure 3B). We noticed that eight RNA expression levels were significantly associated with overall survival outcomes except for CDCA8 and CDC20 (Figures 3C,D). Pathway enrichment analysis results of the 10 hub genes are summarized in Table 2.

**TABLE 2:** Reactome and KEGG pathway enrichment results.

**Term ID**	**Term description**	**Observed counts**	**Background count**	**FDR**
HSA-69278	Cell cycle, mitotic	8	483	8.61E-10
HSA-68886	M phase	6	343	2.74E-07
HSA-68877	Mitotic prometaphase	5	190	7.61E-07
HSA-69620	Cell cycle checkpoints	5	265	3.09E-06
HSA-2500257	Resolution of sister chromatid cohesion	4	118	6.59E-06
HSA-1538133	G0 and early G1	3	27	7.46E-06
HSA-170145	Phosphorylation of proteins involved in the G2/M transition by Cyclin A: Cdc2 complexes	2	3	3.96E-05
HSA-174184	Cdc20: Phospho-APC/C mediated degradation of cyclin A	3	68	8.08E-05
HSA-176408	Regulation of APC/C activators between G1/S and early anaphase	3	76	8.08E-05
HSA-176417	Phosphorylation of Emi1	2	6	8.08E-05
HSA-141444	Amplification of signal from unattached kinetochores via a MAD2 inhibitory signal	3	90	9.66E-05
HSA-2514853	Condensation of prometaphase chromosomes	2	11	0.00013
HSA-1362300	Transcription of E2F targets under negative control by p107 (RBL1) and p130 (RBL2) in complex with HDAC1	2	16	0.00023
HSA-5663220	RHO GTPases activate formins	3	131	0.00023
HSA-174048	APC/C: Cdc20 mediated degradation of cyclin B	2	22	0.00036
HSA-2467813	Separation of sister chromatids	3	178	0.00046
HSA-6804757	Regulation of TP53 degradation	2	35	0.00072
HSA-4615885	SUMOylation of DNA replication proteins	2	40	0.00087
HSA-6791312	TP53 regulates transcription of cell cycle genes	2	49	0.0012
HSA-5688426	Deubiquitination	3	279	0.0013
HSA-597592	Post-translational protein modification	5	1366	0.0013
HSA-69206	G1/S transition	2	128	0.0068
hsa04110 (KEGG)	Cell cycle	3	123	0.00045
hsa05203 (KEGG)	Viral carcinogenesis	3	183	0.00072
hsa04914 (KEGG)	Progesterone-mediated oocyte maturation	2	94	0.0052
hsa04114 (KEGG)	Oocyte meiosis	2	116	0.0059
hsa04218 (KEGG)	Cellular senescence	2	156	0.0084

### Construction of the ceRNA Network in LUAD

**FIGURE 4:**
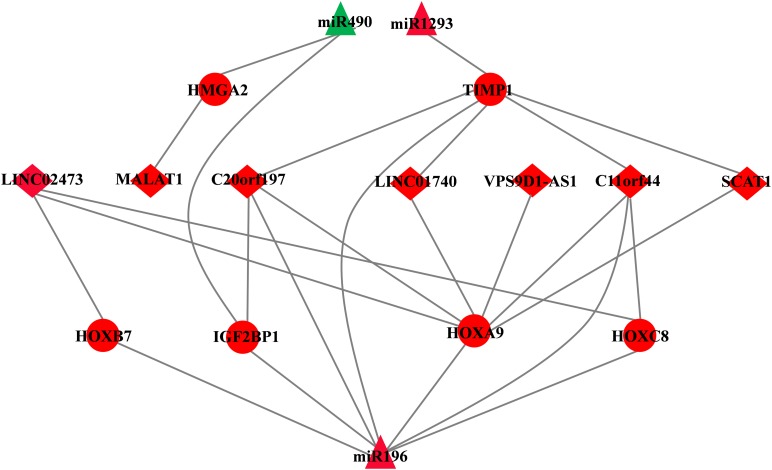
CeRNA network of LUAD. The triangles indicate miRNAs, circles mean mRNAs, and diamonds represent lncRNAs. Red means up-regulated, and green means down-regulated.

A total of seven DElncRNAs, six DEmRNAs, and three DEmiRNAs were enrolled in the ceRNA network (Figure 4). miRTarBase was used to predict the miRNA–mRNA pairs (Table 3). We only focused on those miRNA–mRNA pairs whose interaction evidence was validated by experiments, including luciferase reporter assay, Western blot, Northern blot, or qRT-PCR.

**TABLE 3:** The miRTarBase database revealed interactions between miRNA and mRNAs.

**miRNA**	**mRNA**
miR1293	TIMP1
miR196b	HOXB8, HOXC8, CD8A, HOXA9, MEIS1, FAS, ETS2, RDX, HOXB7, GATA6, TGFBR2, PIK3CG, AKT1, MTOR, FOS, IGF2BP1,
miR490	ERGIC3, FOS, SMARCD1, CCND1, PIK3CA, PAPPA, ABCC2, TGFBR1, HMGA2, TGFA, RHOA, PCBP1, TNKS2, BMPR2, HNRNPA1

Then, we employed a simple linear regression model combined with miRcode database to predict the potential miRNA target by DElncRNAs [see Methods 2.4 model (1)]. In the model, we specified that the input of lncRNAs should be (i) differentially expressed between tumor and normal tissues; (ii) lncRNA expression is associated with overall survival outcomes (logrank *p* value < 0.05). Finally, 7 of 53 DElncRNAs, 6 of 340 DEmRNAs, and 3 of 9 DEmiRNAs formed the ceRNA network. Detailed information about their expression and association with overall survival outcomes is listed in Table 4.

**TABLE 4:** Information about differentially expressed RNAs and miRNAs in ceRNA network.

**DERNAs/DEmiRNAs**	**Log2FC**	***p* value**	**FDR**	**logrank_*p*value**
miR1293	4.00	2.2e-13	1.20e-12	9.48e-05
miR196b	3.94	1.91e-31	2.80e-30	3.45e-02
miR490	−2.31	7.58e-07	2.63e-06	3.53e-02
C20orf197	3.06	9.22e-19	7.80e-18	0.003
SCAT1	2.87	3.25e-23	3.89e-22	0.006
C11orf44	2.32	9.35e-07	2.45e-06	0.019
MALAT1	2.09	5.96e-10	2.22e-09	0.023
VPS9D1-AS1	2.86	3.57e-38	9.82e-37	0.029
LINC02473	4.18	5.18e-25	6.98e-24	0.045
LINC01740	3.32	6.55e-07	1.74e-06	0.049
HMGA2	5.77	1.22e-22	1.39e-21	0.152
TIMP1	1.53	5.33e-25	7.16e-24	0.961
HOXB7	2.09	2.99e-17	2.24e-16	0.006
IGF2BP1	6.41	4.26e-21	4.34e-20	0.042
HOXA9	1.77	1.51e-07	4.33e-07	0.371
HOXC8	2.67	3.23e-13	1.66e-12	0.315

### Screen Biomarkers and Construction Risk Model

**FIGURE 5:**
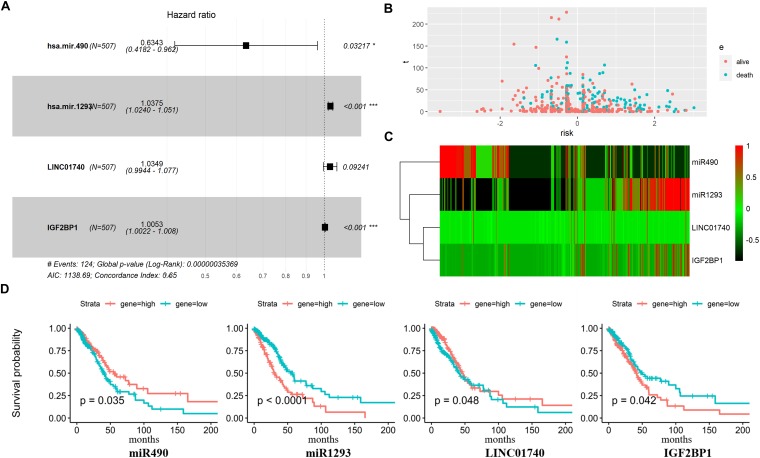
Predictive gene signature analysis. **(A)** Forest map based on the risk score model. Left vertical dotted line indicates protective genes and right risk genes. **(B)** The scatter diagram based on survival time and log2(risk score). The red means alive and green means death. The higher log2(risk score) is, the shorter the time survival. **(C)** Differentially expressed predictive genes that were enrolled in the risk model heatmap. **(D)** Overall survival curves of four predictive genes in LUAD.

Three DEmiRNAs, seven DElncRNAs, and six DEmRNAs in the ceRNA network were selected as candidate biomarkers for the following step analysis. Subsequently, combined univariate Cox regression with a logrank test analysis with *p* value < 0.05 and 12 variables (miR1293, miR196b, miR490, C20orf197, SCAT1, C11orf44, MALAT1, VPS9D-AS1, LINC02473, LINC01740, HOXB7, and IGF2BP1) were identified. Furthermore, a stepwise regression was performed according to the 12 variables. Consequently, four variables including miR490, miR1293, LINC01740, and IGF2BP1 were harvested in the Cox regression. Risk score = −0.455^∗^miR490 + 0.037^∗^miR1293 + 0.034^∗^LINC01740 + 0.005IGF2BP1 (Figure 5A).

Afterward, the LUAD patients were divided into two groups based on the median value of Cox regression model. The distribution of the risk score along with the corresponding survival data and the four protective gene expression demonstrated that the high-risk LUAD patients tended to experience shorter survival time, and low-risk LUAD patients were opposite (Figure 5B). Results show that miR490 and LINC01740 in the high-risk group expression level were lower than that in the low-risk group; meanwhile, miR1293 and IGF2BP1 were opposite (Figures 5C,D).

## Discussion

In this study, a total of 2078 DEmRNAs, 257 DElncRNAs, and 101 DEmiRNAs were identified. GO analysis revealed that the function of DERNAs is mainly associated with receptor ligand activity, ligand-gated ion channel transport, morphogenesis of an epithelium process, and cell–cell adhesion, which play vital roles in tumorigenesis (Valley et al., 2015; Inoue et al., 2016). Biological pathway annotation of DERNAs showed that the GPCR ligand binding process accumulated the largest number of dysregulated genes (86 DERNAs), which indicated that the pathway may play an important role in the development and progression of tumors.

In addition, KEGG pathways analysis showed that DERNAs are mainly enriched in neuroactive ligand–receptor interaction, alcoholism, systemic lupus erythematosus, metabolism of xenobiotics by cytochrome P450, and steroid hormone biosynthesis pathways, which are related to the progression of many cancers, including lung cancer (Ashton et al., 2010; Bulk et al., 2017). Among these enriched pathways, the neuroactive ligand–receptor interaction pathway accumulated the most dysregulated genes (53 DERNAs), indicating that they were associated with lung cancer progression. Multiple DERNAs were enriched in both alcoholism pathway and systemic lupus erythematosus pathway. To our surprise, all DERNAs enriched in these two pathways were up-regulated, except for gene 2354, whose gene symbol is “FOSB.” Gene annotation of FOSB shows that it is a proto-oncogene, and it has been considered as a regulator of cell proliferation, differentiation, and transformation (Liu et al., 2018; Na and Kim, 2018; Park et al., 2019).

In this study, a total of 55 proteins (including 45 DEmRNAs) were enrolled in the PPI network, and pathway enrichment analysis was performed based on the 10 hub genes. Most of the 10 hub genes were associated to cell cycle-related pathways, including M Phase (Zhang et al., 2018; Chung et al., 2019), Cell Cycle Checkpoints (Lee et al., 2016; Wenzel and Singh, 2018), TP53 Regulates Transcription of Cell Cycle Genes (Ni et al., 2018), and Viral carcinogenesis pathways (Villa, 2013; Shibata et al., 2016), which play an important role in occurrence and development of tumors (Florez et al., 2017; Niculescu, 2019). Cell cycle disorder and cell overgrowth are common biological characteristic of tumors, leading to increased cell proliferation and decreased apoptosis (Hsiao et al., 2014; Kebsa et al., 2018). It should be noted that the cell cycle is a tightly regulated process, which is frequently aberrant in lung cancer (Shcherba et al., 2014). By inhibiting the unrestricted cell division and growth of lung cancer cells, cell cycle-related genes have emerged as new targets for the treatment of lung cancer (Girek et al., 2019).

Among the 10 hub genes, 9 were significantly associated with muscle invasive bladder cancer, including CCNA2, CDC20, CDCA8, DLGAP5, KIF20A, NCAPG, NDC80, PBK, and TOP2A (Lee et al., 2012). It indicated that there may exist relative risk between muscle invasive bladder cancer and LUAD. Furthermore, we found that all these 10 hub genes were up-regulated in LUAD tumor tissue. The PPI network showed that almost all the 10 hub genes could interact with each other, and DLGAP5, CDK1, and KIF20A play a key role in connecting the network. Among them, DLGAP5 could physically interact with PBK, TOP2A, and CDK1, and all mitosis-associated proteins correlated with poor prognosis for non-small cell lung cancer patients (Shih et al., 2012; Schneider et al., 2017). In addition, a previous study reported that CCNA2, CDC20, PBK, and TOP2A that interacted with CDK1 play vital roles in survival outcomes in human lung cancer. Loss of cytoplasmic CDK1 could predict poor survival in human lung cancer and confers chemotherapeutic resistance (Zhang et al., 2011). Hence, we concluded that these 10 hub genes play key roles in regulating cell proliferation in LUAD.

A total of three DEmiRNAs, seven DElncRNAs, and six DEmRNAs were enrolled in the ceRNA network. In ceRNA network, we found that MALAT1 as a highly conserved lncRNA whose overexpression has been shown in various cancers, such as breast, prostate, colon, and liver, especially in early stage metastasizing patients (Lin et al., 2007; Guffanti et al., 2009; Xu et al., 2011; Ren et al., 2013). In addition, Ping et al. have reported that MALAT1 can predict metastasis in early stage NSCLC (Ji et al., 2003). Consistent with Ping et al., Lars et al. verified that MALAT1 stimulates migration, invasion, and tumor growth (Schmidt et al., 2011), although the underlying mechanism is poorly understood. In our ceRNA network, the expression of miR490 is down-regulated while MALAT1 and HMGA2 expression is up-regulated in LUAD. One possible explanation is that aberrant expression of MALAT1 acts as a ceRNA for miR-490, and high-expression MALAT1 inhibits miR490 and then increased expression of HMGA2 (the target of miR490), finally accelerating to tumor progression.

Many homeobox genes, including HOXC8, HOXB7, and HOXA9, are also “members” of the ceRNA network. A previous study reported that mis-expression of homeobox genes can lead to abnormal differentiation and proliferation, leading to a change in cell identity or homeotic transformation, therefore playing an important role in carcinogenesis (Samuel and Naora, 2005). In cancer, homeobox genes function as “tumor modulators” as their deregulation normally involve either up-regulation of genes expressed in undifferentiated cells or down-regulation of genes expressed in differentiated tissue, thus acting either as oncogenes or tumor suppressor genes (Abate-Shen, 2002). Almost all the genes in the ceRNA network have reported that they enrolled or associated with tumor progression, except for LINC02473, LINC0170, VPS9D1-AS1, C11orf44, and SCAT1. Hence, taking all these genes in the ceRNA network into consideration, we combined step regression and Cox regression analysis and identified four genes as prognostic biomarkers in LUAD, including miR490, miR1293, LINC01740, and IGF2BP1.

By searching these genes in PubMed, we found that miR490 and IGF2BP1 have been studied for their mechanism in or association with tumor progression. Gain- and loss-of-function studies of miR490 showed that it regulates cell proliferation and is required for induction of *in vitro* migration and invasion (Zhao and Zheng, 2016). miR490 overexpression reduced proliferation, promoted G1 arrest and apoptosis, and suppressed migration and invasion (Sun et al., 2016). In our study, miR490 expression was significantly lower in lung cancer than in normal tissues, and survival analysis result showed that the lower expression miR490 predicted poor survival in lung cancer. Opposite to miR490, IGF2BP1 expression is up-regulated, and the high expression level of IGF2BP1 showed poor overall survival outcomes in lung cancer. Studies reported that IGF2BP1 has been traditionally regarded as an oncogene and potential therapeutic target for cancers (Huang et al., 2018). It plays essential roles in embryogenesis and carcinogenesis, regulating the expression of some essential mRNA targets required for the control of tumor cell proliferation, growth, and invasion, and associating with a poor overall survival and metastasis in various types of human cancers (Gong et al., 2016). However, there is no public report on miR1293 and LINC01740 according to a PubMed search. Univariate Cox regression analysis showed that high expression of miR1293 tended to show poor survival outcomes (logrank_*p*value < 0.0001), and high expression of LINC01740 tended to show good survival outcomes (logrank_value = 0.048). Our results suggest that the four predictive genes may play crucial roles in the pathomechanism of LUAD and act as potential prognostic biomarkers.

Although a four-predictive gene signature was constructed and appears to be potential prognostic biomarkers in clinical application, there are some limitations. First, the prognostic value of LINC01740 is not very satisfactory. Second, the binding affinities between lncRNA and miRNA were predicted by simple linear regression model and miRcode and should be further experimentally investigated. Third, the function and mechanism of the four predictive genes in LUAD need to be further studied by experiments.

In conclusion, we established the disordered ceRNA network, which is beneficial to understanding the relationship among lncRNA–miRNA–mRNA and provides efficient strategies for subsequent functional studies of them. In addition, we identified that miR1293, miR490, LINC01740, and IGF2BP1 might be novel important prognostic factors involved in LUAD pathogenesis, and the risk score model is helpful in studying the overall survival outcome in LUAD.

## Data Availability Statement

The datasets analyzed in this study are publicly available from The Cancer Genome Atlas (TCGA) database, and can be accessed here: https://portal.gdc.cancer.gov/).

## Author Contributions

YW developed the theory and performed the computations. RH verified the analytical methods and results. LM conceived the original idea and supervised the findings of this work. All authors discussed the results and contributed to the final manuscript.

## Conflict of Interest

The authors declare that the research was conducted in the absence of any commercial or financial relationships that could be construed as a potential conflict of interest.

## Footnotes

1 https://portal.gdc.cancer.gov/

2 https://string-db.org/
